# Correction to “Adaptation and Validation of the Indonesian Version of the Problem Areas in Diabetes Scale Among People With Type 2 Diabetes: An Exploratory and Confirmatory Factor Analysis”

**DOI:** 10.1002/nop2.70560

**Published:** 2026-04-15

Fadlilah, S., W. R. Murdhiono, M. Dwidyanti, et al. 2026. “Adaptation and Validation of the Indonesian Version of the Problem Areas in Diabetes Scale Among People With Type 2 Diabetes: An Exploratory and Confirmatory Factor Analysis.” *Nursing Open* 13, no. 3: e70486. https://doi.org/10.1002/nop2.70486.

In the Funding section of the published article, the grant information was incorrectly reported as “This study was funded by The National Science and Technology Council, Taiwan through grant 10.13039/100020595.” This should have read: “This study was funded by the National Science and Technology Council, Taiwan, through grant no. 110‐2314‐B‐038‐081‐MY3 and 113‐2314‐B‐038‐055‐MY3.”

We apologize for this error.

